# Autonomy support, peer relations, and teacher-student interactions: implications for psychological well-being in language learning

**DOI:** 10.3389/fpsyg.2024.1358776

**Published:** 2024-08-21

**Authors:** Di Wu, Xin Dong

**Affiliations:** ^1^Department of French and Francophone Studies, Dalian University of Foreign Languages, Dalian, China; ^2^Education, Training, Work and Knowledge Laboratory, Université Toulouse, Toulouse, France

**Keywords:** teacher-student relationships, autonomy support, peer relationships, psychological well-being, French language learning, structural equation modeling

## Abstract

**Introduction:**

This research explores the intricate interplay among teacher-student relationships, perceived autonomy support, peer relationships, and their collective impact on the psychological well-being of 387 university students enrolled in French language courses across diverse academic institutions in China.

**Methods:**

Employing Confirmatory Factor Analysis (CFA) and Structural Equation Modeling (SEM), this study aims to establish the validity and robustness of the proposed model. Data collection involved online surveys utilizing Likert scales and standardized measures to assess variables concerning educational relationships and psychological well-being.

**Results:**

The findings reveal significant associations between teacher-student relationships, autonomy support, positive peer relationships, and psychological well-being. Importantly, analyses demonstrate the influential role of positive peer relationships in mediating the effects of teacher-student relationships and autonomy support on students’ psychological well-being.

**Discussion:**

These outcomes emphasize the crucial significance of educational relationships in shaping students’ psychological well-being within academic settings. The findings contribute to understanding the nuanced dynamics of educational interactions and their profound implications for student well-being. This highlights the necessity of cultivating positive educational environments for enhanced student mental health.

## Introduction

Language learning within educational settings encompasses a complex web of interactions among students, educators, and peers, exerting profound influences not only on academic achievements but also on students’ psychological well-being (Swain et al., 2002; Philp et al., 2013; Zhang et al., 2022). The dynamics of teacher-student relationships stand as pivotal elements significantly shaping students’ learning experiences and emotional health. These relationships, emphasizing their role in fostering a supportive learning environment and positively affecting students’ emotional well-being (Reeve et al., 2004; Aldrup et al., 2018). Teachers’ provision of autonomy support, a critical aspect encompassing encouragement of students’ independence and initiative within the learning process, has been identified as a key determinant of students’ motivation, engagement, and psychological well-being (Ryan and Deci, 2000; Aldrup et al., 2018; Okada, 2023). Autonomy-supportive teaching practices are known to enhance students’ intrinsic motivation and overall well-being, thereby fostering a positive learning atmosphere (Su and Reeve, 2011).

Moreover, the significance of peer relationships cannot be understated within educational environments. Peer interactions constitute a fundamental component of the educational landscape, profoundly influencing students’ social interactions and overall well-being (Risi et al., 2003; Balluerka et al., 2016). These relationships extend beyond mere socialization; they serve as critical contributors to students’ emotional experiences and psychological health. Positive peer relationships have been associated with increased levels of well-being and reduced feelings of isolation or stress among students (Balluerka et al., 2016; Kuutti et al., 2022). Students’ interactions with their peers shape their sense of belonging, emotional support networks, and overall satisfaction within the academic environment (Strayhorn, 2009; Osterman, 2023).

Despite existing research exploring the individual effects of teacher-student relationships, perceived autonomy support, and peer dynamics on students’ well-being, a thorough examination of their interconnectedness within the context of language learning remains limited. Previous studies might have primarily focused on these variables in isolation, overlooking their potential interdependent relationships and mediation pathways. This study seeks to bridge this gap by exploring the complex network of influences among these variables, specifically within the unique setting of Chinese university students enrolled in French language courses. The significance of focusing on Chinese university students learning French cannot be overstated. China’s growing emphasis on internationalization and its strategic partnerships with Francophone countries have elevated the importance of French language proficiency among university students. This linguistic skill is increasingly seen as vital for careers in international business, diplomacy, and cultural exchange. However, the educational context in which these students learn French is distinct, characterized by high academic expectations, traditional teaching methods, and the unique socio-cultural dynamics of Chinese higher education. These factors create a complex environment that influences students’ academic and psychological experiences in unique ways.

Researching this specific group provides valuable insights into how teacher-student relationships, autonomy support, and peer interactions play out in a setting where students face significant pressures to perform academically while also navigating the challenges of mastering a foreign language. Understanding these dynamics is crucial for developing targeted interventions that can enhance both the academic outcomes and psychological well-being of these students. Additionally, the findings can inform broader educational practices and policies, offering lessons that can be applied in other contexts where students are learning under similar conditions. By examining these relationships simultaneously, this research aims to unravel the mechanisms through which teacher-student relationships and perceived autonomy support influence students’ well-being directly and indirectly through peer interactions. Understanding these interconnected dynamics could offer valuable insights for educators and policymakers to devise interventions and pedagogical strategies that not only enhance academic learning but also foster positive emotional experiences within language education settings. Moreover, by filling this research void, this study aims to contribute significantly to the existing body of knowledge, providing a nuanced understanding of the interplay among these variables and their implications for students’ well-being in language learning contexts.

## Literature review

### Psychological well-being

Psychological well-being constitutes a comprehensive state encompassing emotional, cognitive, and social aspects, reflecting an individual’s mental health and life satisfaction (Ryff, 2013). Its components include emotional resilience, positive affect, self-acceptance, autonomy, and personal growth, not solely representing the absence of distress but encompassing flourishing and thriving in daily life (Keyes, 2005; Huppert, 2009). Within educational domains, psychological well-being assumes considerable importance, profoundly influencing students’ learning outcomes, social interactions, and overall academic success (Suldo and Shaffer, 2008; Huppert and So, 2013; Trudel-Fitzgerald et al., 2019).

Students experiencing heightened well-being tend to actively engage in learning, display enhanced problem-solving skills, and demonstrate greater resilience in academic challenges (Seligman and Csikszentmihalyi, 2000; Li et al., 2019). Moreover, it contributes to a conducive learning environment, fostering a positive school climate and elevating student satisfaction (Durlak et al., 2011). Emotional regulation plays a pivotal role in psychological well-being among students, enabling effective management of emotions crucial in dealing with academic stressors and peer interactions (Brackett et al., 2011). Well-developed emotional regulation skills correlate with reduced anxiety and increased resilience in academic pressures (Brackett and Rivers, 2013).

Self-efficacy beliefs significantly contribute to students’ psychological well-being, representing confidence in their academic capabilities (Bandura, 1997). Higher self-efficacy fosters active engagement in learning tasks, persistence in the face of challenges, and a positive outlook on academic performance (Zimmerman, 2000), promoting competence and mastery in the learning process. Additionally, a sense of belonging within the educational community and supportive relationships with peers and educators exert a substantial impact on students’ psychological well-being (Goodenow, 1993; Jackman et al., 2022). Feeling connected and valued within the academic environment positively influences emotional states, motivation, and overall satisfaction with the educational experience (Anderman, 2003; Li et al., 2019).

Psychological well-being plays a pivotal role in cultivating learning environments conducive to academic achievement and holistic development (Suldo and Shaffer, 2008; Suldo et al., 2011). Higher psychological well-being enhances engagement in learning activities, showcases adaptive coping strategies, and correlates with improved academic performance (Wang and Kanungo, 2004; Suldo, 2016). Moreover, nurturing psychological well-being contributes to a positive school culture, enriching students’ overall educational journey and fostering a sense of fulfillment and personal growth (Bewick et al., 2010).

Several academic inquiries have delved into the intricacies of university students’ mental well-being and the contributory or inhibitory elements affecting their psychological health. Research conducted by Bewick et al. (2010) noted an escalation in the challenges faced by students concerning their mental well-being upon entering university compared to their pre-university phase. Specifically, stress levels were most pronounced during the initial semester but notably subsided as students progressed from the first to the subsequent semester across various academic years. Burris et al. (2009) outlined several determinants linked to the psychological well-being and distress of students. They established a positive correlation between higher psychological well-being and lower distress with traits such as optimism, health values, and religious inclinations. Conversely, spirituality and an increased number of sexual partners were associated with diminished well-being. In a different vein, Harding et al. (2019) examined the correlation between educators’ mental health and the well-being of their students. Their findings revealed a significant link between depressive symptoms exhibited by teachers and the decreased well-being and psychological distress experienced by students. They indicated that teacher mental health holds a substantive sway over student outcomes, highlighting the potential influence of teacher presenteeism and the quality of teacher-student relationships in this dynamic.

Furthermore, Kardas et al. (2019) conducted an investigation into the predictors of well-being. They emphasized gratitude, hope, optimism, and life satisfaction as pivotal factors that forecast an individual’s psychological well-being, emphasizing their fundamental role in nurturing positive mental health outcomes. Collectively, these studies underscore the multifaceted nature of elements that impact the psychological well-being of university students. These encompass a spectrum of individual traits, contextual influences, dynamics within the teacher-student relationship, and personal attitudes and viewpoints.

### Peer relationships

Peer relationships represent a critical facet of students’ experiences within educational settings, playing a profound role in socio-emotional development, academic engagement, and overall well-being (Wentzel, 2017). The influence of positive peer relationships extends beyond mere social connections, fostering a sense of belonging, support, and social integration that create an environment conducive to learning and personal growth (Wentzel, 1998, 2016). Crucially, peers serve as vital sources of social support, offering companionship, encouragement, and empathy that significantly impact students’ emotional well-being (Brown and Larson, 2009; Riese et al., 2012). The research evidence underscores that students perceiving their peer relationships positively often exhibit lower levels of stress, anxiety, and loneliness, contributing positively to their psychological well-being (Chan et al., 2016).

Moreover, peer relationships wield considerable influence on students’ academic achievement and motivation. Constructive peer interactions can stimulate learning through collaborative activities, peer tutoring, and exchanging constructive feedback (Hughes and Chen, 2011; Wentzel, 2016). The extant literature indicate that students engaged in supportive peer networks tend to showcase heightened motivation, increased academic aspirations, and improved academic performance (Wentzel and Watkins, 2002). Beyond academics, peers significantly shape students’ behaviors, attitudes, and decision-making processes. Social comparison and modeling within peer groups influence various academic behaviors like study habits, classroom engagement, and attitudes towards learning (Wentzel and Watkins, 2002; Wentzel, 2016). Additionally, peer interactions impact students’ perceptions of the school climate, teacher-student relationships, and their overall school experience (Wentzel and Caldwell, 1997).

However, despite the benefits, challenges such as peer conflict, bullying, and social exclusion can adversely affect students’ well-being and academic engagement (Rudolph, 2010). Interventions aimed at peer mediation, conflict resolution programs, and cultivating empathy have displayed potential in alleviating negative peer interactions and fostering a positive peer culture within educational institutions (Allen and Loeb, 2015). Efforts focused on peer support programs, such as peer mentoring or buddy systems, have proven effective in enhancing peer relationships and establishing a supportive school environment (DeRosier et al., 2013; Watson, 2017). These programs facilitate positive interactions, offer guidance, and promote inclusivity among students, contributing to a heightened sense of community and belonging within the school environment.

Several studies have illuminated the influential impact of peer connections on students’ psychological well-being across varied educational contexts and developmental stages. Armsden and Greenberg’s (1987) exploration of adolescent parent and peer attachment revealed a correlation between secure peer attachments and positive psychological well-being. Their findings accentuated how the quality of adolescents’ peer relationships significantly affected their emotional health and overall well-being. In a different vein, Hanson et al. (2016) delved into the influence of peer learning on psychological well-being within higher education settings. Their conclusions highlighted the positive impact of collaborative learning experiences among peers on students’ psychological well-being, underscoring the role of such environments in nurturing students’ mental health. Balluerka et al. (2016) scrutinized the determinants of adolescents’ psychological well-being, focusing on peer attachment and class emotional intelligence. Their study unveiled that robust peer attachments and higher emotional intelligence within class settings correlated with enhanced psychological well-being among adolescents, emphasizing the supportive influence of peer relationships on emotional health.

Furthermore, Buchanan and Bowen (2008) investigated the impact of peer support in conjunction with adult support on the psychological well-being of middle-school students. Their findings indicated that peer support significantly contributed to students’ overall well-being, emphasizing the pivotal role of peer relationships alongside adult support in fostering positive psychological outcomes among students. Another study by Kibret and Tareke (2017) centered on the contribution of instructor, peer, and university support in bolstering psychological well-being among university students. Their research demonstrated that peer support emerged as a significant predictor of students’ psychological well-being, spotlighting the influential role of peer relationships in the university milieu. Moreover, Oberle et al. (2010) explored the nexus between social and emotional well-being and peer relations during early adolescence. Their discoveries identified gender-specific predictors of peer acceptance, showcasing the significance of positive peer relations in fostering social and emotional well-being during this developmental stage.

In sum, these studies collectively underscore the pivotal function of peer relationships in shaping students’ psychological well-being across diverse educational levels. Positive peer relationships, characterized by attachment, support, and collaborative interaction, emerge as influential elements in nurturing students’ emotional health and overall psychological well-being during adolescence and within educational environments.

### Teacher autonomy support

Teacher autonomy support, a cornerstone of effective pedagogy, encompasses educators’ acknowledgment and respect for students’ perspectives, choices, and self-initiation in the learning journey (Reeve and Jang, 2006; Su and Reeve, 2011). This approach aims to nurture a sense of competence, ownership, and intrinsic motivation among students, significantly influencing their motivation, engagement, and academic performance (Núñez et al., 2015; Reeve, 2016). It stands as a pivotal factor fostering students’ active involvement and enhancing their learning experiences (Guay, 2022). The practices associated with autonomy support in teaching revolve around offering students meaningful rationales, acknowledging their viewpoints, and providing opportunities for decision-making and independent problem-solving within the learning context (Patall et al., 2008; Reeve and Cheon, 2021). It is argued that that educators fostering autonomy support cultivate environments where students feel valued, competent, and motivated to participate actively in learning tasks, creating a conducive space for growth and development (Reeve, 2016; Reeve and Cheon, 2016).

The impact of autonomy-supportive teaching on students’ motivation and learning outcomes has been substantial. The extant literature underscores that exposure to autonomy-supportive teachers correlates with higher levels of intrinsic motivation, persistence, and deeper engagement in academic tasks. This support instills a sense of volition and control, leading to improved conceptual understanding and higher-quality learning outcomes (Reeve et al., 2004; Reeve and Cheon, 2016). Moreover, teacher autonomy support significantly contributes to students’ psychological well-being within educational settings (Jiang and Tanaka, 2022). It is evident that when students feel autonomy, relatedness, and competence in their learning environment, it positively influences their emotional state, school satisfaction, and overall well-being (Soenens and Vansteenkiste, 2010; Neufeld and Malin, 2020). This link between autonomy support and psychological well-being highlights its significance beyond academic achievement, emphasizing its role in nurturing students’ holistic development. However, despite its evident advantages, implementing autonomy-supportive teaching practices can present challenges. Teachers often encounter obstacles stemming from curriculum demands, standardized assessments, or traditional teaching approaches that hinder their ability to effectively provide autonomy support (Reeve and Cheon, 2021). To overcome these challenges, professional development programs focusing on enhancing teachers’ skills in fostering autonomy support are essential (Assor, 2017).

Creating autonomy-supportive environments extends beyond individual teacher practices; it requires a school-wide culture that values autonomy, collaboration, and student agency in the learning process (Deci et al., 2017; Cho et al., 2022). Collaborative efforts among teachers, administrators, and policymakers are fundamental in fostering a systemic shift towards autonomy-supportive educational environments, thereby ensuring a comprehensive and enriching learning experience for students (Conklin, 2013). Numerous studies have shed light on the pivotal influence of perceived instructor autonomy support in shaping students’ psychological well-being across diverse educational environments. Jiang and Tanaka (2022) conducted a study examining the autonomy support offered by support personnel in higher education, revealing a strong association between this support and students’ academic engagement and psychological well-being. Their results emphasized that autonomy support from various educational staff, beyond classroom teachers, significantly impacted the overall well-being of students. Neufeld and Malin (2020) delved into the perceptions of instructor autonomy support among medical students and its mediation of their motivation and psychological well-being. Their research highlighted how autonomy support significantly fostered motivation and well-being in medical students, indicating its potential to positively influence students’ psychological health in specialized academic domains.

A longitudinal investigation by Kleinkorres et al. (2023) focused on students’ well-being development during adolescence and underscored the role of perceived instructor autonomy support. Their study accentuated that students’ perceptions of autonomy support from their teachers wielded a lasting effect on their well-being over time, highlighting the enduring impact of teacher autonomy support on students’ psychological health. Collie and Martin (2017) examined teachers’ adaptability and its connection to perceived autonomy support, teachers’ well-being, and students’ numeracy achievement. Their findings suggested a positive relationship between teachers’ adaptability, perceived autonomy support, and students’ psychological well-being. It indicated that teachers fostering autonomy support positively influenced students’ well-being and academic outcomes. Moreover, Gutiérrez and Tomás (2019) explored the function of perceived support for autonomy in anticipating the academic achievement of university students. This prediction was mediated through academic self-efficacy and engagement in school activities.

Their study underscored the substantial impact of autonomy support in fostering academic self-efficacy and school engagement, indirectly contributing to students’ academic success and overall well-being.

Also, several studies have explored how perceived teacher autonomy support impacts peer relationships among students across educational settings. Zhao and Qin (2021) found that perceived autonomy support influenced students’ self-efficacy, moderated by perceived peer support, revealing a complex interaction between teacher support and peer dynamics in shaping students’ learning experiences. Ruzek et al. (2016) examined how teacher emotional support motivated students, showing that it positively impacted students’ perceptions of peer relatedness and autonomy support. This emphasized how positive teacher-student relationships foster a supportive environment, enhancing students’ perceptions of their peer relationships. Conducted research focusing on the influence of perceived autonomy support from peers, parents, and physical education instructors on the physical activity and health-related quality of life in adolescents. Their findings indicated a significant predictive relationship between perceived autonomy support from peers and both physical activity levels and health-related quality of life, highlighting the role of peer relationships influenced by perceptions of autonomy support in promoting adolescents’ well-being.

Overall, these collective studies emphasize the critical role of perceived instructor autonomy support in bolstering students’ psychological well-being across diverse educational settings. The findings suggest that when students perceive autonomy support from their educators, it positively influences their engagement, motivation, academic accomplishments, and overall psychological well-being within educational contexts.

### Teacher-student relationships

Teacher-student relationships encompass the emotional bonds and interpersonal connections forged between educators and their students within the academic setting (Pianta et al., 2005; Roorda et al., 2017). These relationships are characterized by trust, mutual respect, and supportive interactions, holding immense significance due to their profound impact on students’ socio-emotional growth, academic engagement, and overall well-being (Rudasill et al., 2010; Longobardi et al., 2016). Research emphasizes the pivotal role of relationship quality, linking positive teacher-student connections to numerous favorable outcomes (Fabris et al., 2023). The related research has highlighted the correlation between high-quality relationships and students’ motivation, engagement in learning, and social competence (Pianta et al., 2012; Longobardi et al., 2021).

The influence of teacher-student relationships extends notably to academic achievement (Martin and Collie, 2019). A positive and supportive rapport marked by warmth and encouragement correlates strongly with higher academic success (Pianta and Stuhlman, 2004; Roorda et al., 2011). Students who perceive their teachers as caring and supportive tend to exhibit increased academic engagement, better classroom behavior, and improved performance academically (Lee, 2012; Quin, 2017). Beyond scholastic outcomes, these relationships significantly impact students’ emotional well-being, acting as a protective factor against emotional distress. Supportive teacher-student relationships contribute positively to students’ psychological well-being by reducing stress and anxiety levels (Skinner et al., 2009; Murray-Harvey, 2010; Holfve-Sabel, 2014).

The quality of teacher-student relationships is influenced by multifaceted factors encompassing both teacher and student aspects (Pianta and Stuhlman, 2004; Chan et al., 2023). Teacher-related factors like empathy, communication skills, and effective classroom management interact with student-related elements such as socio-emotional development, behavioral traits, and cultural backgrounds to shape these relationships (Buyse et al., 2008; Sun and Shi, 2022). Longitudinal studies highlight the enduring impact of early-established teacher-student connections on students’ academic and social development, underscoring the importance of continuity in fostering these positive relationships throughout their educational journey (Pianta et al., 2005). Nevertheless, challenges may arise in nurturing positive teacher-student relationships due to diverse student needs, classroom dynamics, or constraints in teacher resources (Murray and Malmgren, 2005). Nevertheless, interventions focusing on professional development, relationship-building strategies, and cultivating positive classroom environments have shown promise in enhancing these connections (MacSuga-Gage et al., 2012). Investing in teacher training programs emphasizing relationship-building skills, cultural understanding, and empathy can significantly enhance educators’ abilities to establish and maintain positive connections with their students (Bryk and Schneider, 2002; Li et al., 2022). These interventions play a crucial role in fostering positive teacher-student relationships, which in turn significantly impact students’ academic achievement, socio-emotional well-being, and long-term development within educational settings (Hagenauer et al., 2015).

The impact of teacher-student connections on students’ mental wellness within educational environments has been well-documented in numerous studies. Aldrup et al. (2018) explored the connection between student misconduct and the well-being of instructors, unveiling that the quality of teacher-student relationships acted as a mediator. Their research highlighted the potential enhancement of teacher well-being through fostering positive engagements with students, indirectly influencing student well-being. Similarly, Lin et al. (2022) examined the influence of closeness in student-teacher relationships on students’ psychological well-being, focusing on hope as a mediating element. Their findings suggested that stronger student-teacher connections fostered greater hope among students, ultimately benefiting their psychological well-being.

Poulou (2020) scrutinized students’ school adjustment concerning teachers’ need satisfaction and teacher-student relationships. The study revealed that positive relationships with teachers significantly contributed to students’ overall adjustment and well-being in the school setting. Zheng’s (2022) research underscored the significance of teacher interpersonal behavior in impacting students’ well-being. The study indicated that positive teacher interpersonal behavior mediated students’ well-being, emphasizing the role of teacher-student relationships in fostering a supportive environment for students’ psychological welfare. Moreover, Murray-Harvey (2010) investigated the influence of relationships on students’ academic performance, mental health, and general well-being. Their results reiterated the profound impact of positive relationships, highlighting that these connections not only affected academic achievements but also played a crucial role in students’ mental health and well-being. Finally, Holfve-Sabel (2014) delved into student well-being components, emphasizing learning, interaction, and relationships. These components were identified as crucial factors contributing to student well-being, underscoring the significance of pleasant teacher-student relationships in providing a supportive and enriching learning context conducive to students’ psychological welfare.

Also, some studies highlight the relationship between teacher-student dynamics and peer relationships in diverse educational contexts. Endedijk et al. (2022) conducted a meta-analysis, revealing that the quality of teacher-student relationships significantly influences peer relationships among students, emphasizing the pivotal role of teachers in shaping positive peer interactions. Uslu and Gizir (2017) investigated adolescents’ school belonging, finding that positive teacher-student relationships significantly contributed to students’ sense of belonging, indicating a strong link between these relationships and the formation of positive peer connections in schools. Zhu et al. (2022) explored resilience, teacher-student relationships, and peer dynamics. Their study highlighted that positive relationships with teachers were associated with improved peer relationships and greater resilience, aiding in alleviating mental health difficulties among students.

Taken together, these studies collectively emphasize how teacher-student relationships impact peer dynamics. Positive teacher-student connections not only benefit student well-being but also foster supportive and inclusive peer interactions within schools. They also underscore the substantial influence of teacher-student relationships on students’ psychological well-being within educational settings. Positive and supportive relationships between teachers and students emerge as pivotal factors significantly contributing to students’ overall psychological health and well-being in educational contexts.

### Educational landscape of Chinese universities for students learning French

The educational landscape of Chinese universities presents a unique context for students pursuing French language studies. With China’s growing emphasis on internationalization and cross-cultural exchange, learning French has gained increasing popularity among university students (Huang, 2003). This shift is partly driven by China’s expanding diplomatic and economic relations with Francophone countries, which have created a demand for proficiency in the French language in various professional sectors, including international business, diplomacy, and tourism (Bianco, 1988). In Chinese universities, French language programs are typically housed within the broader framework of foreign language departments or dedicated French studies departments. These programs offer a range of courses that cover language proficiency, literature, culture, translation, and applied linguistics (Zhang, 2020). The curriculum is designed to provide students with a comprehensive understanding of the French language and Francophone cultures, aiming to equip them with the skills necessary for both academic and professional success (Gong et al., 2020).

Chinese universities emphasize a student-centered approach to language learning, integrating various pedagogical strategies to enhance student engagement and motivation. These strategies often include interactive classroom activities, language immersion experiences, and the use of multimedia resources (McCafferty, 2006). Additionally, many universities have established partnerships with French institutions, facilitating student exchanges, internships, and collaborative research projects (Jiang and Shen, 2019). These initiatives provide students with valuable opportunities to immerse themselves in Francophone environments, thereby enhancing their linguistic and cultural competence (Wang et al., 2013). The role of the teacher in Chinese universities is particularly significant in shaping students’ learning experiences. Teachers are expected to provide not only academic instruction but also emotional and motivational support. This dual role aligns with the principles of autonomy support, where teachers encourage students to take initiative and responsibility for their learning while providing the necessary guidance and encouragement (Gyeltshen and Gyeltshen, 2022). The quality of teacher-student relationships is thus a critical factor influencing students’ academic and psychological outcomes (Ma et al., 2018).

Peer interactions also play a crucial role in the educational experiences of Chinese students learning French. Collaborative learning activities, peer tutoring, and group projects are commonly employed to foster a supportive learning community (Pan and Yuan, 2023). These peer interactions contribute to the development of social networks that offer emotional support, enhance language practice opportunities, and promote a sense of belonging within the academic environment (Yang, 2024).

Despite the numerous advantages, students learning French in Chinese universities may face several challenges. These include high academic expectations, pressure to achieve proficiency quickly, and balancing language studies with other academic responsibilities (Wright and Zheng, 2016). Moreover, the transition from traditional rote learning methods to more interactive and student-centered approaches can be challenging for both students and educators (Li and Cutting, 2011). Overall, the educational landscape of Chinese universities provides a dynamic and supportive environment for students learning French. The interplay of teacher support, peer relationships, and institutional resources creates a fertile ground for fostering psychological well-being and academic success. Understanding this context is essential for interpreting the findings of the present study and for developing targeted interventions to further enhance the educational experiences of Chinese university students pursuing French language studies (Zhang, 2016).

### The structural model

Understanding the interplay between teacher-student relationships, perceived teacher autonomy support, peer relationships, and psychological well-being is pivotal in comprehending the holistic educational experiences of Chinese university students studying French. This section elucidates and supports four hypotheses examining the direct and mediated relationships between these variables, drawing upon established theoretical frameworks and empirical evidence.

**H1**: Teacher–student relationship is directly related to the psychological well-being of Chinese university students of French.

The Self-Determination Theory (SDT) emphasizes that positive teacher-student relationships contribute significantly to students’ psychological well-being (Deci and Ryan, 2000). Empirical evidence in educational settings consistently supports this assertion. Aldrup et al. (2018) demonstrated that positive teacher-student relationships are linked with higher psychological well-being in students. Lin et al. (2022) reiterated this relationship within the Chinese educational context. Studies by Poulou (2020), Mohammad Hosseini et al. (2022), and Zheng (2022) also confirm that robust teacher-student relationships positively impact students’ psychological well-being across diverse educational levels and cultural settings.

Moreover, within language learning environments, Henry and Thorsen (2018) and Derakhshan et al. (2022) specifically highlight the pivotal role of supportive teacher-student relationships in fostering emotional health and engagement in learning a second language. These studies collectively reinforce the notion that positive teacher-student relationships are fundamental for enhancing the psychological well-being of students studying French in Chinese universities.

**H2**: Perceived teacher autonomy support is directly related to the psychological well-being of Chinese university students of French.

Theoretical frameworks like SDT underscore the significance of perceived autonomy support from teachers in nurturing students’ psychological well-being (Deci and Ryan, 2000). Empirical research further validates this relationship. Gutiérrez and Tomás (2019), Jiang and Tanaka (2022), and Kleinkorres et al. (2023) affirm the positive correlation between perceived teacher autonomy support and students’ psychological well-being in various educational contexts.

O'Reilly (2014) specifically within language education underscores the role of perceived autonomy support from teachers in influencing students’ motivation, engagement, and overall well-being in language learning. These findings support the assertion that perceived teacher autonomy support is directly associated with the psychological well-being of Chinese university students studying French.

**H3**: Peer relationship quality mediates the relationship between teacher–student relationship and psychological well-being of the students.

Evidence from studies such as Uslu and Gizir (2017), Endedijk et al. (2022), and Zhu et al. (2022) suggests a significant impact of positive teacher-student relationships on peer relationships among students. Additionally, Buchanan and Bowen (2008), Hanson et al. (2016), and Kibret and Tareke (2017) underscore the link between positive peer relationships and students’ psychological well-being across diverse educational contexts. Therefore, it is plausible to suggest that the quality of teacher-student relationships indirectly influences students’ psychological well-being through the mediation of peer relationships, as these relationships are interlinked and mutually influential in shaping students’ overall well-being.

**H4**: Peer relationship quality mediates the relationship between perceived teacher autonomy support and psychological well-being of the students.

Similarly, empirical findings from Ruzek et al. (2016), Tilga et al. (2021), and Zhao and Qin (2021) indicate that perceived autonomy support from teachers can influence peer relationships. Further, Armsden and Greenberg (1987), Oberle et al. (2010), and Balluerka et al. (2016) highlight the significance of positive peer relationships in contributing to students’ psychological well-being. Hence, considering the reciprocal influence between perceived teacher autonomy support, peer relationship quality, and psychological well-being, it is conceivable that perceived teacher autonomy support indirectly impacts students’ psychological well-being through the mediation of peer relationship quality.

In summary, these hypotheses are firmly rooted in established theoretical frameworks such as SDT, supported by extensive empirical studies across educational contexts. They underscore the critical roles of teacher-student relationships, perceived teacher autonomy support, and peer relationships in shaping the psychological well-being of Chinese university students studying French.

## Materials and methods

### Participants and procedures

The study involved 387 Chinese university students enrolled in French language courses across 12 academic institutions located in multiple provinces in China. Participants, aged between 18 and 25 years, showcased a diverse age distribution, with approximately 47% falling within the 20–22 age bracket, around 28% in the 18–19 age group, and roughly 25% aged between 23 and 25. Gender distribution within the participant pool demonstrated a balanced representation, encompassing approximately 202 female students and 185 male students, resulting in a nearly equal ratio of 52% female and 48% male participants. In terms of French language proficiency, the cohort showcased a varied range of proficiency levels: approximately 74 students (19%) identified as beginners, around 220 (57%) as intermediate learners, and roughly 93 (24%) as advanced learners. This distribution facilitated a comprehensive representation of proficiency levels among the study participants.

Data collection procedures exclusively utilized quantitative methods, employing online surveys disseminated through university communication channels and language course platforms. The survey instruments incorporated Likert scales and standardized measures to assess variables pertinent to teacher-student relationships, perceived autonomy support, peer dynamics, and psychological well-being. The six-week data collection window was designed to accommodate participants’ academic schedules, ensuring ample time for responses. Participants were approached through official university email lists, course forums, and dedicated online platforms, providing convenient access to the survey materials. Engagement in the study was voluntary, and strict adherence to ethical standards ensured that informed consent was acquired from each participant before their involvement.

### Instruments

#### Teacher-student relationship

The assessment of the teacher-student relationship utilized a survey adapted from the Teacher-Student Relationship Scale (TSRS) developed by Pianta and Nimetz (1991), which was originally designed to assess the relationships between kindergarten children and their teachers. To ensure the appropriateness of this measure for college students, the TSRS was modified to suit the Chinese cultural context and the higher education environment by Wang and Wang (2002). The adapted survey comprised 15 items aimed at gauging three dimensions: closeness, positive reactivity, and conflict. The participants rated their head teacher’s behaviors on a 5-point Likert scale ranging from 1 (strongly disagree) to 5 (strongly agree). Sample items include: “My teacher makes me feel important,” “My teacher responds positively to my needs,” and “My teacher and I often have conflicts.” Scores were computed by averaging responses across items, where higher scores indicated more favorable teacher-student relationships. To establish the validity of the adapted TSRS for college students, a Confirmatory Factor Analysis (CFA) was conducted, demonstrating good model fit with the data (χ^2^ = 312.56, df = 132, *p* < 0.001, CFI = 0.93, TLI = 0.91, RMSEA = 0.05). The reliability of the scale was assessed using Cronbach’s alpha, which showed high internal consistency for the overall scale (α = 0.83) and for each subscale: closeness (α = 0.85), positive reactivity (α = 0.83), and conflict (α = 0.79).

#### Peer relationship

Evaluation of peer relationships employed the Peer Relationship Quality Scale for Adolescents, developed by Bae et al. (2015). This scale encompassed positive and negative peer relationship factors, with a total of 13 items. Eight items assessed positive peer relationships, while five items evaluated negative peer relationships. Notably, the negatively phrased items were reversed during analysis. Sample items include: “I feel supported by my peers” and “I often have disagreements with my peers.” The scale demonstrated strong reliability with a reported Cronbach’s alpha of 0.85 in the original study by Bae et al. (2015). For the current study, the reliability was re-assessed and showed a Cronbach’s alpha of 0.89. CFA results indicated good construct validity (χ^2^ = 228.45, df = 102, p < 0.001, CFI = 0.92, TLI = 0.90, RMSEA = 0.06).

#### Teacher autonomy support

This study utilized the autonomy support scale designed by Shih (2013), adapted from the original scale by Williams and Deci (1996). The scale comprised six items assessing students’ perceptions of Teacher Autonomy Support (TAS) during classes. Responses were collected on a 6-point Likert scale ranging from ‘fully disagree’ (1) to ‘fully agree’ (6). Sample items include: “My teacher provides me with choices and options,” and “My teacher encourages me to take initiative in my learning.” The Cronbach’s alpha for the TAS scale in the current study was 0.91, indicating high reliability. The CFA confirmed the scale’s validity with satisfactory fit indices (χ^2^ = 45.67, df = 15, p < 0.001, CFI = 0.96, TLI = 0.94, RMSEA = 0.05).

#### Psychological well-being

Psychological well-being was assessed using the shortened version of the Psychological Well-being Scale (Ryff and Keyes, 1995). This 18-item scale encompassed six facets: autonomy, environmental mastery, personal growth, positive relations, purpose in life, and self-acceptance. Each facet consisted of three items measured on a 7-point scale, reversed for consistency in scoring. Sample items include: “I feel in charge of the situation in which I live,” and “I have a sense of direction and purpose in life.” The reliability of the scale was high, with a Cronbach’s alpha of 0.86. CFA results showed good model fit (χ^2^ = 271.38, df = 120, p < 0.001, CFI = 0.94, TLI = 0.92, RMSEA = 0.05), confirming the validity of the measure for the current study.

### Data analysis

Data analysis was initiated using SPSS 28.0 for preliminary descriptive statistics and correlation assessments. Subsequently, Confirmatory Factor Analysis (CFA) was conducted using AMOS 26.0 (Arbuckle, 2017) to assess construct validity and model fitness. This analysis aimed to establish the expected associations between latent constructs and observable indicators, ensuring alignment between the proposed model and the collected data. Following the CFA, Structural Equation Modeling (SEM) was employed to explore the assumed relationships among latent constructs.

In evaluating model fit, several commonly used fit indices were considered, including the χ2/df ratio, Tucker-Lewis Index (TLI), Comparative Fit Index (CFI), Root-Mean-Square Error of Approximation (RMSEA), and Standardized Root-Mean-Square Residual (SRMR) (Hair et al., 2014). According to Hu and Bentler (1999), criteria for acceptable model fit are more stringent. Specifically, a good model fit is indicated by a χ2/df ratio close to 2, TLI and CFI values ≥0.95, RMSEA values ≤0.06, and SRMR values ≤0.08. Additionally, to ensure the reliability of indirect effects, bootstrapping analyses employing 5,000 resamples were conducted (Hayes, 2017).

## Results

### Initial analyses

Prior to conducting statistical analyses, various data pre-processing steps were undertaken to ensure dataset integrity (Field, 2013). Assessments for normality included skewness, kurtosis indices, histograms, and Q-Q plots. Variables exhibiting substantial deviations from normal distribution underwent transformations to meet assumptions required for statistical tests and mitigate potential biases stemming from non-normality (Tabachnick and Fidell, 2013). Specifically, the variables measuring peer relationship quality and teacher autonomy support displayed significant positive skewness and kurtosis. Consequently, log transformations were applied to these variables to normalize their distributions. Additionally, the variable assessing psychological well-being showed slight negative skewness and underwent square root transformation for normalization.

Addressing missing data was a priority, guided by Little’s Missing Completely at Random (MCAR) test (Little, 1988), which indicated randomness in the missingness pattern (χ^2^ = 245.17, df = 231, *p* = 0.234). The missing data rates for the variables were as follows: teacher-student relationship (2.1%), peer relationship quality (3.4%), teacher autonomy support (1.8%), and psychological well-being (2.6%). To handle these missing values effectively, multiple imputation techniques were applied, generating 20 imputed datasets (Graham, 2012). This approach ensured robustness in subsequent analyses and maximal retention of available information, allowing for more reliable and valid results.

Outliers underwent thorough scrutiny employing both univariate and multivariate outlier analyses (Tabachnick and Fidell, 2013). Univariate outliers were identified based on z-scores and boxplots for each variable, flagging extreme values beyond ±3 standard deviations from the mean. Multivariate outliers were assessed using Mahalanobis distance (Tabachnick and Fidell, 2013), targeting cases with unusual combinations of multiple variables. Detected outliers were examined for accuracy and outliers impacting data integrity were corrected or removed accordingly.

### Descriptive statistics analysis

Descriptive statistics and correlations among the key constructs—teacher-student relationship, autonomy support, peer relationship, and psychological well-being—are presented in Table 1. The mean scores for teacher-student relationship, autonomy support, peer relationship, and psychological well-being were 3.46 (SD = 0.73), 3.71 (SD = 0.69), 3.93 (SD = 0.73), and 3.42 (SD = 0.88), respectively. Reliability analysis using Cronbach’s alpha demonstrated acceptable internal consistency for each construct: teacher-student relationship (α = 0.83), autonomy support (α = 0.91), peer relationship (α = 0.89), and psychological well-being (α = 0.86).

**Table 1:** Descriptive statistics and correlations among the constructs.

Variables	Mean	SD	Cronbach’s α	1	2	3	4
1.Teacher-student relationship	3.46	0.73	0.83	1			
2. Autonomy support	3.71	0.69	0.91	0.35^**^	1		
3. Peer relationship	3.93	0.73	0.89	0.51^***^	0.45^***^	1	
4. Well-being	3.42	0.88	0.86	0.42^***^	0.39^**^	0.58^***^	1

** *p* < 0.01.

*** *p* < 0.001.

Correlational analyses revealed significant associations among the variables. A moderately positive correlation was found between autonomy support and teacher-student relationship (*r* = 0.35, *p* < 0.01), indicating a relationship between teachers’ provision of autonomy support and the quality of their relationships with students. Additionally, both autonomy support and peer relationship exhibited significant positive correlations with each other (*r* = 0.45, *p* < 0.001), suggesting a link between students’ perceived autonomy support and the quality of their peer relationships within the educational setting.

Furthermore, correlations with psychological well-being revealed significant associations. Teacher-student relationship exhibited a moderate positive correlation with psychological well-being (*r* = 0.42, *p* < 0.001), indicating that higher levels of positive teacher-student relationships were associated with enhanced psychological well-being among language learners. Conversely, both autonomy support (*r* = 0.39, *p* < 0.01) and peer relationship (*r* = 0.58, *p* < 0.001) displayed moderate to strong positive correlations with psychological well-being, indicating that higher levels of perceived autonomy support and positive peer relationships were linked to increased psychological well-being among students.

### Measurement model testing

The initial phase of analysis involved scrutinizing the measurement model using CFA to assess construct validity and model fit. The CFA aimed to verify the proposed measurement model’s adequacy by examining the associations between latent constructs and their respective observed indicators.

The measurement model exhibited satisfactory fit indices, endorsing the adequacy of the specified latent constructs. The chi-squared value for the measurement model was χ^2^(310) = 815.30, with a χ^2^/df ratio of 2.63, within the recommended threshold of 3, indicating a reasonable model fit (*p* < 0.001). Additionally, the TLI and CFI values were 0.92 and 0.94, respectively, surpassing the criterion of 0.90. The RMSEA was 0.067 (CI 90%: 0.058–0.076), lower than the cutoff of 0.08, suggesting a close fit to the data. Moreover, the SRMR value was 0.075, adhering to the threshold of 0.10, signifying an acceptable model fit (Hu and Bentler, 1999). The standardized factor loadings for all indicators on their respective latent constructs were statistically significant (*p* < 0.001) and above the recommended threshold of 0.50, ranging from 0.62 to 0.85. The constructs demonstrated discriminant validity, as the square roots of the Average Variance Extracted (AVE) were higher than the inter-construct correlations, affirming that constructs shared more variance with their respective indicators than with other constructs (Fornell and Larcker, 1981).

Table 2 showcases the AVE and CR values, fundamental indices for assessing these two types of validity. The AVE values for each construct—teacher-student relationship (0.52), autonomy support (0.63), peer relationship (0.58), and psychological well-being (0.66)—all surpassed the recommended threshold of 0.50 (Fornell and Larcker, 1981). These findings indicate that more than 50% of the variance in the indicators is explained by their respective constructs, affirming convergent validity (Hair et al., 2014). The CR values for all constructs—ranging from 0.83 to 0.91—surpassed the conventional threshold of 0.70, denoting high internal consistency reliability (Fornell and Larcker, 1981). This suggests that the latent constructs reliably capture the variance of their respective indicators, supporting convergent validity (Hair et al., 2014).

**Table 2:** Convergent and divergent validity.

Variables	AVE	CR	1	2	3	4
1.Teacher-student relationship	0.52	0.91	**0.72**			
2. Autonomy support	0.63	0.86	0.35^**^	**0.79**		
3. Peer relationship	0.58	0.83	0.51^***^	0.45^***^	**0.76**	
4. Well-being	0.66	0.89	0.42^***^	0.39^**^	0.58^***^	**0.81**

AVE = Average Variance Extracted, CR = Composite Reliability. bold font numbers are square roots of the AVE; off diagonals are correlation confidents.

** *p* < 0.01,

*** *p* < 0.001.

Moreover, to establish divergent validity, interconstruct correlations were examined (Fornell and Larcker, 1981). The off-diagonal values in Table 2 demonstrate the correlations between constructs. Notably, while significant positive correlations were found among constructs (e.g., between autonomy support and teacher-student relationship), these correlations were below the squared AVE values for each construct (Fornell and Larcker, 1981). For instance, the correlation between autonomy support and teacher-student relationship (r = 0.35) was lower than both constructs’ AVE squared (0.52 and 0.63, respectively), indicating support for divergent validity.

These results collectively suggest that the measurement model exhibits adequate Convergent and Divergent Validity, confirming that the indicators measure their respective constructs effectively and that each construct is distinct from the others.

### The SEM analysis

**Figure 1:**
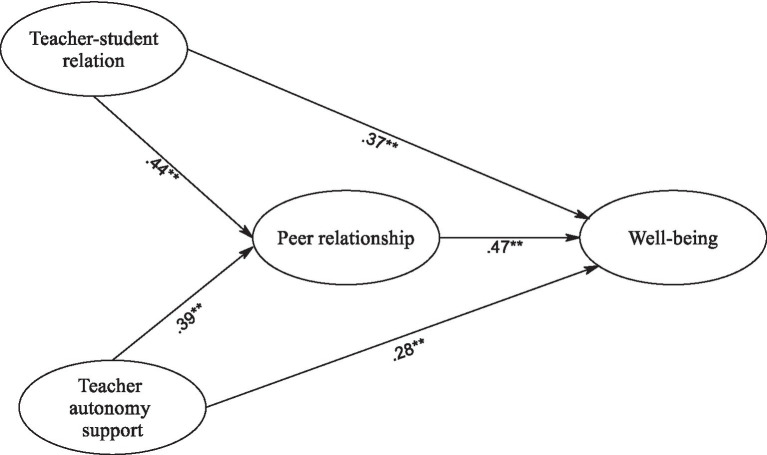
The SEM Model. ^*^p < 0.01, ^**^p < 0.001.

Consequently, we employed SEM to explore the hypothesized connections among the latent constructs. Our scrutiny of model fit indices revealed a consistent alignment between the postulated model and the amassed data, indicating an acceptable fit with χ^2^(311) = 530, CFI = 0.968, TLI = 0.957, RMSEA = 0.031 [CI 95%: 0.027–0.036], SRMR = 0.035. In Figure 1, a visual representation of the hypothesized relationships among the latent constructs is presented, demonstrating the statistical significance of all path coefficients. These findings provide substantial support for the expected associations between the variables. Moreover, to assess the significance of indirect effects, we conducted bootstrapping analyses utilizing 5,000 resamples, following the methodology described by Hayes (20177). Table 3 encapsulates a concise summary of the bootstrapping outcomes, encompassing direct, indirect, and total effects within the mediation analysis.

**Table 3:** The SEM results.

Path	β	Bootstrapped 95% CI	*p*
Direct effects			
T-S relationship → Well-being	0.37	[0.25, 0.47]	<0.001
Autonomy support → Well-being	0.28	[0.18, 0.40]	<0.001
Peer relationship → Well-being	0.47	[0.38, 0.56]	<0.001
Indirect effects			
T-S relationship → Peer relationship → Well-being	0.20	[0.12, 0.28]	<0.01
Autonomy support → Peer relationship → Well-being	0.18	[0.09, 0.25]	<0.01
Total effects			
T-S relationship → Well-being	0.57	[0.45, 0.67]	<0.001
Autonomy support → Well-being	0.46	[0.35, 0.57]	<0.001

T-S relationship = teacher-student relationship, Autonomy support = Teacher autonomy support.

As seen in Table 3, the direct effects revealed significant associations between the latent constructs and psychological well-being. Specifically, the teacher-student relationship exhibited a positive association with psychological well-being (*β* = 0.37, 95% CI [0.25, 0.47], *p* < 0.001), indicating that a stronger teacher-student relationship corresponded to higher levels of well-being among students. Similarly, autonomy support displayed a positive relationship with well-being (*β* = 0.28, 95% CI [0.18, 0.40], *p* < 0.001), as did peer relationship (*β* = 0.47, 95% CI [0.38, 0.56], *p* < 0.001). These findings suggest that greater autonomy support and positive peer relationships contribute significantly to students’ psychological well-being.

The indirect effects, delineating the mediated pathways, also exhibited noteworthy associations. The indirect path from teacher-student relationship to well-being through peer relationship displayed a significant positive relationship (*β* = 0.20, 95% CI [0.12, 0.28], *p* < 0.001). Similarly, the indirect path from autonomy support to well-being via peer relationship indicated a significant positive relationship (*β* = 0.18, 95% CI [0.09, 0.25], *p* < 0.001). These outcomes imply that both teacher-student relationship and autonomy support influence students’ well-being partially through the mediation of positive peer relationships.

When considering the total effects, encompassing both direct and indirect pathways, substantial associations were observed. The cumulative effect of teacher-student relationship on well-being (total effect: *β* = 0.57, 95% CI [0.45, 0.67], *p* < 0.001) and autonomy support on well-being (total effect: *β* = 0.46, 95% CI [0.35, 0.57], *p* < 0.001) remained significantly positive. These results highlight the considerable impact of teacher-student relationship and autonomy support on students’ psychological well-being, combining both direct and mediated influences.

## Discussion

The primary aim of this study was to investigate the intricate relationships among teacher-student interactions, perceived teacher autonomy support, peer dynamics, and the psychological well-being of Chinese university students enrolled in French language courses. Through an exploration of these relationships, the study sought to elucidate the pathways through which teacher-student and teacher-autonomy support interactions impact students’ well-being, both directly and indirectly via peer relationships. The empirical investigation aimed to contribute to the existing body of literature by providing insights into the mediating roles of peer relationships in the association between teacher support systems and students’ psychological well-being within the unique context of language education at Chinese universities.

Firstly, a direct relationship was found between perceived teacher autonomy and psychological well-being of Chinese university students of French. It is notably consistent with Self-Determination Theory (SDT), a framework emphasizing autonomy as a core psychological need crucial for optimal functioning and well-being (Ryan and Deci, 2000). SDT underscores autonomy as the sense of volition and choice individuals experience in their actions, allowing them to align their behaviors with their values, interests, and preferences. Within an educational setting, when students perceive autonomy support from their instructors—such as encouragement to voice opinions, make choices regarding learning activities, or exert control over their learning process—they experience a heightened sense of ownership and control (Neufeld and Malin, 2020). This, in turn, satisfies their innate need for autonomy and cultivates intrinsic motivation (Deci and Ryan, 2000). This finding harmonizes with prior research emphasizing the influence of autonomy-supportive environments on psychological well-being (Collie and Martin, 2017; Gutiérrez and Tomás, 2019; Neufeld and Malin, 2020; Jiang and Tanaka, 2022; Kleinkorres et al., 2023). In language instruction, when instructors provide choices, validate students’ perspectives, and involve them in decision-making regarding their learning journey, it positively impacts their psychological well-being and engagement in language learning tasks (O'Reilly, 2014).

Furthermore, autonomy-supportive teaching practices not only elevate students’ psychological well-being but also contribute to their academic success and overall satisfaction with the learning process (Gutiérrez and Tomás, 2019; Jiang and Tanaka, 2022; Kleinkorres et al., 2023). Across various educational contexts, studies consistently show that autonomy support from teachers is linked to higher motivation levels, increased engagement, and enhanced satisfaction among students (Núñez et al., 2015; Gutiérrez and Tomás, 2019; Kleinkorres et al., 2023). Moreover, SDT asserts that autonomy support augments positive emotional experiences and psychological wellness by bolstering feelings of competence and connectedness, complementing the fundamental need for autonomy (Deci and Ryan, 2000). In the realm of language learning, where students grapple with linguistic barriers and cultural disparities, the provision of autonomy support from teachers gains particular significance. In the context of French language learning, perceiving autonomy in their learning environment—such as having control over their learning pace or involvement in decision-making—could alleviate stress, bolster self-efficacy, and positively impact emotional well-being (He, 2009). This nurturing of autonomy could potentially act as a buffer against challenges, fostering a more positive emotional experience and enhancing students’ well-being as they navigate the intricacies of learning a new language.

Secondly, a direct relationship was found between teacher-student relationships and the psychological well-being of Chinese university students. This finding aligns with research grounded in the theoretical perspective of attachment theory (Bowlby, 1988), which emphasizes the pivotal role of secure and supportive relationships in nurturing emotional well-being and resilience (Karreman and Vingerhoets, 2012). Originally developed to explain the bonds between children and their primary caregivers, attachment theory has been extensively applied to various relational contexts, including educational settings. Research has shown that the principles of attachment theory are highly relevant to adolescent psychology, as the quality of relationships with significant adults, such as teachers, continues to play a crucial role in students’ emotional and psychological development during this period (O’Connor and McCartney, 2007; Riley, 2010). Within educational contexts, positive teacher-student relationships facilitate students’ emotional regulation, mitigating stress and anxiety while contributing significantly to their psychological well-being (Poulou, 2020; Lin et al., 2022). Adolescents benefit from supportive teacher relationships as they navigate increased academic pressures and social dynamics. Studies have found that adolescents with secure and positive relationships with their teachers are more likely to experience higher levels of school engagement, better academic performance, and improved mental health outcomes (Allen et al., 2011; Roorda et al., 2011).

The observed direct correlation between teacher-student relationships and the psychological well-being of Chinese university students aligns with existing literature (Murray-Harvey, 2010; Holfve-Sabel, 2014; Aldrup et al., 2018; Zheng, 2022). These studies demonstrate that students perceiving positive relationships with their teachers exhibit elevated emotional well-being, reduced stress levels, and improved mental health outcomes (Murray-Harvey, 2010; Rudasill et al., 2010; Poulou, 2020; Lin et al., 2022). Positive teacher-student relationships are also associated with amplified positive affect, diminished depressive symptoms, and an overall enhancement in students’ psychological well-being (Zheng, 2022). Furthermore, these relationships significantly influence academic engagement and success. Positive teacher-student relationships correlate with heightened student engagement, motivation, and academic achievement (Roorda et al., 2011; Hagenauer et al., 2015). Students who feel connected to their teachers are more motivated in their learning endeavors, actively participate in classroom activities, and demonstrate improved academic performance (Holfve-Sabel, 2014). This underscores the importance of nurturing positive teacher-student relationships for both emotional well-being and academic success.

Thirdly, peer relationship quality mediated the pathway from teacher-student relationship to psychological well-being. This finding is supported by robust empirical evidence and theoretical frameworks emphasizing the interdependence between these relational dynamics (Uslu and Gizir, 2017; Endedijk et al., 2022; Zhu et al., 2022). Past research consistently highlights that positive teacher-student relationships foster an environment conducive to influencing peer interactions among students (Uslu and Gizir, 2017; Endedijk et al., 2022; Zhu et al., 2022). Similarly, positive peer relationships have been strongly associated with students’ psychological well-being (Armsden and Greenberg, 1987; Buchanan and Bowen, 2008; Oberle et al., 2010; Balluerka et al., 2016; Hanson et al., 2016; Kibret and Tareke, 2017). Hence, it is plausible that the impact of teacher-student relationships on psychological well-being operates through the mediation of peer relationship quality. Positive teacher-student relationships cultivate a supportive classroom climate that influences students’ social interactions and peer relationships. Consequently, these positive peer interactions significantly contribute to students’ emotional health and overall psychological well-being (Hanson et al., 2016).

This finding draws from established theories such as social support theories, ecological systems theory, and the socio-ecological model to provide a comprehensive understanding of the interaction among these relationships (Bronfenbrenner, 1979; House et al., 1988). Social support theories emphasize the significance of various social networks, illustrating how these networks can mutually influence each other (Cohen and Wills, 1985). Positive teacher-student relationships act as a primary social support system within the school environment. When students develop supportive and positive connections with their teachers, it fosters feelings of security, trust, and emotional support, ultimately contributing to their psychological well-being (Hamre and Pianta, 2001). Moreover, ecological systems theory and the socio-ecological model underscore the multiple levels of influence on an individual (Bronfenbrenner, 2000), including microsystems (like teacher-student relationships) and mesosystems (involving interactions between different microsystems such as teacher-student and peer relationships). When students experience positive interactions with their teachers, it positively shapes their social–emotional development, social skills, and relational competence (Fabris et al., 2023). These enhanced social competencies subsequently influence the quality of their peer relationships (Zhu et al., 2022). Students who experience supportive and positive relationships with their teachers tend to exhibit higher social competence, engage in more prosocial behaviors, and demonstrate better cooperation skills (Aldrup et al., 2018). These enhanced attributes contribute significantly to fostering more positive and mutually supportive interactions among peers.

Finally, our study revealed that peer relationship quality mediated the interaction between perceived teacher autonomy support and learners’ well-being. This mediation is comprehensively examined within the SDT framework, which posits that environments supportive of autonomy, competence, and relatedness enhance psychological well-being (Deci and Ryan, 2000). Perceived teacher autonomy support aligns with SDT by emphasizing student choice, acknowledging student perspectives, and nurturing competence (Reeve and Jang, 2006). Empirical research supports that perceived autonomy support from teachers fosters feelings of competence and autonomy, positively influencing students’ psychological well-being (Collie and Martin, 2017; Gutiérrez and Tomás, 2019; Neufeld and Malin, 2020; Kleinkorres et al., 2023). Students perceiving teacher support in decision-making and action initiation experience heightened intrinsic motivation and psychological well-being (Jiang and Tanaka, 2022).

Notably, the quality of peer relationships mediates this effect. Positive teacher-student relationships significantly shape students’ social–emotional development, enhancing social skills and relational competence (Roorda et al., 2011). Students perceiving autonomy support from teachers demonstrate higher social competence, engage in prosocial behaviors, and exhibit better cooperation skills (Skinner et al., 2009). Moreover, students experiencing autonomy support from teachers are more likely to develop supportive peer relationships, fostering a sense of relatedness and encouraging positive social interactions (Uslu and Gizir, 2017; Zhu et al., 2022). Perceived teacher autonomy support promotes empowerment and self-regulation, leading to positive peer interactions (Ruzek et al., 2016; Tilga et al., 2021). Consequently, positive peer relationships in autonomy-supportive environments significantly contribute to students’ emotional health and overall well-being (Ruzek et al., 2016). Thus, perceived teacher autonomy support indirectly influences students’ psychological well-being through its impact on peer relationship quality. The supportive, autonomous environment facilitated by teachers directly enhances well-being and indirectly shapes it by fostering positive peer interactions. This underscores the critical role of autonomy-supportive teaching practices in enhancing students’ intrinsic motivation and psychological well-being by improving peer relationship quality within educational settings.

## Conclusion and implications

In summary, this research delved deeply into the intricate interactions between teacher-student relationships, perceived teacher autonomy support, peer dynamics, and the well-being of Chinese university students enrolled in French language courses. The findings underscore the significance of these interconnected factors within the educational milieu and their implications for students’ emotional health. The identification of mediation pathways elucidates the crucial role of positive teacher-student interactions and autonomy-supportive teaching approaches in fostering not only students’ well-being directly but also indirectly through the cultivation of supportive peer relationships.

An important consideration in interpreting these findings is the potential variation in stress levels across different stages of university education. Research indicates that stress levels can fluctuate significantly from the freshman year to the final year of university, often peaking during transitions such as the first year and periods of intense academic pressure, such as exams or thesis preparation (Burris et al., 2009; Bewick et al., 2010). This variation in stress levels may influence how students perceive and benefit from teacher-student relationships, autonomy support, and peer interactions. For instance, first-year students might experience higher levels of stress due to the adjustment to university life, making supportive teacher-student relationships and autonomy-supportive practices particularly crucial during this period. Conversely, senior students, who may face stress related to career planning and final academic requirements, might benefit more from well-established peer support networks that provide both academic assistance and emotional support. Therefore, the effectiveness of these supportive relationships and practices may vary depending on the students’ year of study and specific stressors they face at different stages.

The findings of this study carry substantial implications for educational practices and future research endeavors. Firstly, the identified mediation effects underscore the importance of fostering positive teacher-student relationships and autonomy-supportive teaching approaches within language education settings. Educators should aim to create classroom environments that prioritize supportive interactions, allowing students to feel empowered and encouraged in their learning journey. By emphasizing autonomy-supportive practices, teachers can indirectly facilitate the development of positive peer relationships among students, which in turn, positively influence students’ psychological well-being. These implications emphasize the potential for educators to become agents in cultivating not only academic growth but also emotional support networks for students within the language learning context. Moreover, these findings suggest the need for targeted interventions focusing on enhancing peer relationships as a means to augment students’ psychological well-being. Implementing strategies that promote collaborative learning, peer cooperation, and mutual respect among students could serve as catalysts for fostering supportive peer networks. Furthermore, creating opportunities for peer group activities and discussions may fortify these relationships, leading to positive emotional experiences and well-being among students engaged in French language courses. Tailoring these interventions to address the specific stressors and needs of students at different stages of their university education could enhance their effectiveness, ensuring that all students receive the support they need throughout their academic journey.

However, several limitations should be acknowledged when interpreting the study findings. The cross-sectional nature of the research design limits the establishment of causal relationships among the variables investigated. Longitudinal studies would provide a more comprehensive understanding of the temporal sequences and directionality of effects, offering insights into the sustainability of the identified relationships over time. Additionally, the study’s focus on Chinese university students enrolled in French language courses may restrict the generalizability of the findings to other educational settings or language learning contexts. Therefore, caution should be exercised in extrapolating these findings to broader populations or diverse linguistic disciplines. Finally, while this study explored the mediation effects, other potential variables or contextual factors influencing the relationships between teacher-student dynamics, peer relationships, and psychological well-being remain unexplored, suggesting avenues for further research exploration and elucidation.

## Data Availability

The raw data supporting the conclusions of this article will be made available by the authors, without undue reservation. Requests to access these datasets should be directed to DW, 15842641833@163.com.
