# Supplementary material for: Autonomy support, peer relations, and teacher-student interactions: implications for psychological well-being in language learning
Source: Front Psychol. 2024 Aug 21;15:1358776. doi: 10.3389/fpsyg.2024.1358776 (PMC11412206; doi:10.3389/fpsyg.2024.1358776)
Supplement: Supplementary file 2 [file Data_Sheet_1.PDF]

Informed Consent Form

Title of Study: *Autonomy Support, Peer Relations, and Teacher-Student Interactions: Implications for Psychological Well-being in Language Learning*

Investigator(s):

- Di Wu (Corresponding Author), Department of French and Francophone Studies, Dalian University of Foreign Languages, Dalian 16044, China Email: 15842641833@163.com
- Xin Dong, Education, Training, Work and Knowledge Laboratory, University Toulouse 2, Toulouse, 31100, France Email: dxin241@gmail.com

Introduction

You are invited to participate in a research study conducted by Di Wu and Xin Dong. The purpose of this study is to explore the complex relationships between teacher-student interactions, perceived autonomy support, peer relationships, and their impact on the psychological well-being of university students enrolled in French language courses across various academic institutions in China.

Procedures

If you agree to participate, you will be asked to complete an online survey. The survey will involve questions related to your educational experiences, relationships with teachers and peers, as well as questions regarding your psychological well-being. The survey will utilize Likert scales and standardized measures to gather information. Your participation will take approximately [estimated time] to complete.

Confidentiality and Privacy

All information collected during this study will be kept confidential and anonymous. Your responses will only be used for research purposes and will be reported in aggregate form. Your identity will be protected, and your personal information will not be disclosed to anyone outside the research team.

Voluntary Participation and Right to Withdraw

Your participation in this study is voluntary. You have the right to refuse to participate or withdraw from the study at any time without penalty or consequence. Your decision will not affect your relationship with the researchers or your academic institution.

Contact Information

If you have any questions about the study, please contact:

- Di Wu at 15842641833@163.com

Consent

I have read the above information and understand the nature of the research study. I have had the opportunity to ask questions and have received satisfactory answers. I voluntarily agree to participate in this research study.

Participant's Name: _________________________________

Participant's Signature: ______________________________ Date: ________________

Researcher's Name: ______________________________ Date: ________________
