# Supplementary material for: Autonomy support, peer relations, and teacher-student interactions: implications for psychological well-being in language learning
Source: Front Psychol. 2024 Aug 21;15:1358776. doi: 10.3389/fpsyg.2024.1358776 (PMC11412206; doi:10.3389/fpsyg.2024.1358776)
Supplement: Supplementary file 3 [file Data_Sheet_2.pdf]

General Instructions for Questionnaire

Dear Participant,

Thank you for taking the time to participate in our research study. Your contribution is invaluable to us. Please carefully read and respond to the following items. Your responses will be used solely for research purposes and will remain confidential.

Demographics Information:

Name (Optional): [__________]

Gender: [ ] Male (1)   [ ] Female (2)
Age Group: [ ] 18-19 (1)   [ ] 20-22 (2)   [ ] 23-25 (3)
French Proficiency: [ ] Beginners (1) [ ] Intermediate (2)   [ ] Advanced (3)

Please ensure that you carefully read and consider each item before marking your response.

Your participation is highly appreciated.

Thank you.

Teacher-Student Relationship Scale

Please reflect on the degree to which each of the following statements currently applies to your relationship with your head teacher.

(Please select the number that best represents your perception.)

1=Definitely does not apply
2=Not really
3=Neutral, not sure
4=Applies somewhat
5=Definitely applies

Items

1. My teacher makes me feel important.
2. My teacher is supportive of me.
3. I feel comfortable sharing my feelings with my teacher.
4. My teacher understands my needs.
5. My teacher responds positively to my needs.
6. My teacher encourages me to succeed.
7. I receive praise from my teacher for good work.
8. My teacher acknowledges my efforts in class.
9. My teacher and I often have conflicts.
10. My teacher sometimes feels frustrated with me.
11. I feel that my teacher is often critical of me.
12. There are frequent disagreements between my teacher and me.
13. My teacher makes me feel valued as a student.
14. My teacher listens to my concerns.
15. My teacher is fair and unbiased in their treatment of students.

Teacher Autonomy Support Scale

Please rate the extent to which you agree or disagree with each statement below.

1=Strongly Disagree
2=Disagree
3=Somewhat Disagree
4=Somewhat Agree
5=Agree
6=Strongly Agree

Items

1. I feel that my teacher provides me choices and options.
2. I feel that my teacher encourages me to take initiative in my learning.
3. I feel a lot of trust in my teacher.
4. My teacher answers my questions fully and carefully.
5. I feel that my teacher cares about me as a person.
6. I feel able to share my feelings with my teacher.

Peer Relationship Scale

Please indicate the extent to which you agree or disagree with each statement regarding your relationships with your friends.

Responses were collected on a 6-point Likert scale ranging from 'fully disagree' (1) to 'fully agree' (6).

1. I spend time with my friends.
2. Friends share their sadness and difficulties with me.
3. I feel supported by my peers.
4. I can talk about my secrets to my friends.
5. When I do something, my friends help me.
6. Friends like me and follow me well.
7. Friends are interested in me.
8. I have a good relationship with my friends.
9. I often have disagreements with my peers.
10. If I fight with a friend, we don't make up easily.
11. If a friend behaves differently from what I want, I get angry or annoyed.
12. I don't want to get close to kids who are different from me.
13. My friends are not interested in my difficulties and hardships.

Psychological Wellbeing Scale

Please indicate the extent to which you agree or disagree with each statement about yourself.

Responses were collected on a 7-point Likert scale ranging from: 1 (Strongly disagree); 2 (Disagree), 3 (A little disagree), 4 (Neither agree nor disagree), 5 (A little agree); 6 (Agree); 7 (Strongly agree)

1. I like most parts of my personality.
2. When I look at the story of my life, I am pleased with how things have turned out so far.
3. Some people wander aimlessly through life, but I am not one of them.
4. The demands of everyday life often get me down.
5. In many ways, I feel disappointed about my achievements in life.
6. Maintaining close relationships has been difficult and frustrating for me.
7. I live life one day at a time and don't really think about the future.
8. In general, I feel I am in charge of the situation in which I live.
9. I am good at managing the responsibilities of daily life.
10. I have a sense of direction and purpose in life.
11. For me, life has been a continuous process of learning, changing, and growth.
12. I think it is important to have new experiences that challenge how I think about myself and the world.
13. People would describe me as a giving person, willing to share my time with others.
14. I gave up trying to make big improvements or changes in my life a long time ago.
15. I tend to be influenced by people with strong opinions.
16. I have not experienced many warm and trusting relationships with others.
17. I have confidence in my own opinions, even if they are different from the way most other people think.

18. I judge myself by what I think is important, not by the values of what others think is important.
