# Supplementary material for: Autonomy support, peer relations, and teacher-student interactions: implications for psychological well-being in language learning
Source: Front Psychol. 2024 Aug 21;15:1358776. doi: 10.3389/fpsyg.2024.1358776 (PMC11412206; doi:10.3389/fpsyg.2024.1358776)
Supplement: Supplementary file 4 [file Data_Sheet_3.PDF]

Study Protocol

Title: *Autonomy Support, Peer Relations, and Teacher-Student Interactions: Implications for Psychological Well-being in Language Learning*

Principal Investigators:

Di Wu (Corresponding Author)

Department of French and Francophone Studies,

Dalian University of Foreign Languages,

Dalian 16044, China

Email: 15842641833@163.com

Xin Dong

Education, Training, Work and Knowledge Laboratory,

University Toulouse 2, Toulouse, 31100, France

Email: dxin241@gmail.com

Background and Rationale:

The study aims to examine the impact of teacher-student relationships, perceived autonomy support, and peer relations on the psychological well-being of Chinese university students enrolled in French language courses. This research addresses the necessity to understand educational interactions and their implications for student mental health within diverse academic settings.

Objectives:

- Evaluate the associations between teacher-student relationships, autonomy support, peer dynamics, and psychological well-being.
- Assess the potential mediating role of positive peer relationships in the impact of teacher-student relationships and autonomy support on psychological well-being.

Participants and Procedures:

The study involves Chinese university students aged between 18 and 25 enrolled in French language courses across various academic institutions in China. Data collection utilized online surveys distributed through university communication channels. Participation was voluntary, and

informed consent was obtained from each participant. The data collection period accommodated participants' academic schedules.

Instruments:

Teacher-Student Relationship: Utilized a survey adapted from Pianta and Nimetz (1991), modified to suit the Chinese cultural context.

Peer Relationship: Employed the Peer Relationship Quality Scale for Adolescents (Bae et al., 2015).

Teacher Autonomy Support: Used the autonomy support scale designed by Shi (2009), adapted from Williams and Deci (1996).

Psychological Well-being: Employed a shortened version of the Psychological Well-being Scale (Ryff & Keyes, 1995).

Data Analysis:

Preliminary descriptive statistics and correlations were planned to be conducted using SPSS 28.0. Confirmatory Factor Analysis (CFA) in AMOS 26.0 was intended to establish construct validity. Structural Equation Modeling (SEM) was planned to explore relationships among constructs. Model fit indices, including $\chi 2/df$, TLI, CFI, RMSEA, and SRMR, were to be considered for fit assessment. Bootstrapping analyses (5000 resamples) were intended to ensure the reliability of indirect effects.

Ethical Considerations:

The study adheres to ethical standards. Plans for obtaining informed consent, participant confidentiality, and anonymity have been established. The research respects individual rights and follows the guidelines of the Declaration of Helsinki.

Ethics Committee Approval:

This study protocol has been submitted to the Ethics Committee of Dalian University of Foreign Languages for review and approval. All necessary documentation, including informed consent forms and study instruments, has been provided for ethical evaluation.
